# Grid-Based Bayesian Filtering Methods for Pedestrian Dead Reckoning Indoor Positioning Using Smartphones

**DOI:** 10.3390/s20185343

**Published:** 2020-09-18

**Authors:** Miroslav Opiela, František Galčík

**Affiliations:** Institute of Computer Science, Faculty of Science, Pavol Jozef Šafárik University, Jesenná 5, 041 54 Košice, Slovakia; frantisek.galcik@upjs.sk

**Keywords:** indoor positioning, smartphone, PDR, Bayes filter, advanced point-mass, grid-based filter

## Abstract

Indoor positioning systems for smartphones are often based on Pedestrian Dead Reckoning, which computes the current position from the previously estimated location. Noisy sensor measurements, inaccurate step length estimations, faulty direction detections, and a demand on the real-time calculation introduce the error which is suppressed using a map model and a Bayesian filtering. The main focus of this paper is on grid-based implementations of Bayes filters as an alternative to commonly used Kalman and particle filters. Our previous work regarding grid-based filters is elaborated and enriched with convolution mask calculations. More advanced implementations, the centroid grid filter, and the advanced point-mass filter are introduced. These implementations are analyzed and compared using different configurations on the same raw sensor recordings. The evaluation is performed on three sets of experiments: a custom simple path in faculty building in Slovakia, and on datasets from IPIN competitions from a shopping mall in France, 2018 and a research institute in Italy, 2019. Evaluation results suggests that proposed methods are qualified alternatives to the particle filter. Advantages, drawbacks and proper configurations of these filters are discussed in this paper.

## 1. Introduction

Pedestrian navigation in a building complex [1], guiding visually impaired visitors in a museum [2], helping patients to find a ward in a hospital [3], navigating a drone in a warehouse [4], positioning in a historical building [5], navigating a person on a wheelchair [6], orientating firefighters in a unknown indoor environment [7], and navigating cars in a parking garage [8] describe application examples of indoor navigation system including the indoor localization. The existence of various use cases determines assorted requirements on a positioning system. Unlike the outdoor navigation, there is no unique adopted solution, as GNSS signal (e.g., GPS) is generally not available indoors.

Typically, an indoor localization system available to a large scale of users utilizes the smartphones with embedded sensors. As an alternative for some use cases, standalone Inertial Measurement Unit (IMU), consisting of accelerometers, gyroscopes, and magnetometers, is fixed on a human body, mostly on the foot [9] or sensors are attached to a robot [10]. Smartphone-based implementations able to cope with the sensor bias are demanded, as the sensors produce noisy and inaccurate measurements [11,12,13]. Unlike the robot navigation, a smartphone position may be less predictable leading to approaches for an activity recognition [14,15] to distinguish a movement type (e.g, walking, standing, using elevators, or escalators) or a smartphone placement (e.g., in hand, in a pocket, or near ear in calling mode). These methods improve the location awareness and provide additional input for the localization system. Typically, methods for the classification problem of human activity recognition are based on machine learning approaches, mostly artificial neural networks, e.g., long short-term memory (LSTM) [16].

Some positioning approaches require an infrastructure in the building often including its calibration and maintenance, e.g., solutions based on Bluetooth Low-Energy (BLE) devices [3], Ultra-wideband (UWB) [17], existing Wi-Fi access points [18], or so-called pseudolites to transmit signals detectable by GPS receivers [19]. On the other hand, an infrastructure-independent approach called Pedestrian Dead Reckoning (PDR) [9] exploits human kinematics and incorporates processed sensor measurements as detected steps, their headings, and a map model into the relative position estimation. The technique is suitable for a fusion with other methods, e.g., PDR, Wi-Fi, and landmarks (walk types) [20], or PDR and visual landmarks (lights) [21]. A Bayesian filtering probabilistically estimates the system state and is able to deal with the uncertainty introduced by noisy measurements making the PDR approach applicable.

In this paper, we consider a use case where a user is equipped with a smartphone with embedded sensors. Precise floor plans are available and no additional building infrastructure or devices are required. Evaluation experiments are situated in a faculty building, a research institute, and a shopping mall. The main aim of the paper is to evaluate different implementations of the Bayesian filtering, analyze the results depending on selection of their parameter values, and compare them with focus on the localization accuracy.

This paper is organized as follows. In Section 2, a related work based on Bayesian filtering and PDR is reviewed followed by the overview of our approach and comments on the Bayesian filtering applied on indoor positioning. In Section 3, the basic grid-based filter [22] is referenced and extended with the convolution mask calculation, and further elaborated to a so-called centroid grid filter. Section 4 introduces the advanced point-mass filter which we applied on the indoor positioning. This filter is able to reduce some drawbacks of other grid-based approaches. The evaluation (Section 5) reveals observations in three buildings where the algorithms are analyzed offline with different parameter configurations on the same measurements. Moreover, the evaluation is performed using various configurations of the particle filter to provide a reference for other methods, and the paper is concluded with the results discussion and recommendations for the parameters setting.

## 2. Solution Background and Related Work

The dead reckoning approach computes a current user or device position from the previously estimated position. Considering pedestrians, it is called pedestrian dead reckoning (PDR). Bayes filters probabilistically estimate a state of a dynamic system using noisy measurements obtained up to the current time of the estimation [23]. The Bayesian approach has found numerous applications in various fields. In [24], a list of selected domains is proposed including, but not limited to, target tracking, computer vision, robotics, speech enhancement and recognition, machine learning, financial and time series analysis, and fault diagnosis. The Bayesian filtering and the PDR are two core components of the proposed system. The PDR approach estimates a new user location and the Bayes filtering technique incorporates the map model and deals with the uncertainty caused by noisy measurements and inaccuracies introduced by PDR, e.g., inaccurate step length estimation.

### 2.1. Pedestrian Dead Reckoning and Bayesian Filtering Formulation

The PDR method calculates a position relative to the current estimation. When a step is detected, the succeeding location is calculated from the sensors measurements, which may be expressed as follows,

(1) $${\mathrm{position}}_{t}={\mathrm{position}}_{t-1}+L_{t}{\left[sin\left(\theta_{t}\right),cos\left(\theta_{t}\right)\right]}^{T}$$

where $\theta_{t}$ is the heading and $L_{t}$ is the length of the step detected at the time *t*.

Imperfect smartphone sensors producing noisy measurements are not capable of indicating the accurate state (considering the indoor positioning, it is the user position and possibly other parameters, e.g., the direction or the velocity). The uncertainty introduced by measurements is modeled by Bayes filter, which represents the state at the time k∈N by a multivariate random variable $x_{k}$. The belief is a probability distribution over $x_{k}$. The aim of the filter is to sequentially estimate the conditional probability density function (pdf) $p(x_{k}|z_{1:k})$ of the state $x_{k}$ given the sensor data as measurements $z_{1:k}=\left\{z_{i},i=1,\cdots ,k\right\}$ for the discrete-time stochastic system:

(2) $$x_{k}=f_{k}\left(x_{k-1}\right)+w_{k},\;k=1,2,\cdots$$

(3) $$z_{k}=h_{k}\left(x_{k}\right)+v_{k},\;k=1,2,\cdots$$

where $x_{k}\in R^{n_{x}}$ is a vector representing the system state and $z_{k}\in R^{n_{z}}$ is a vector representing the measurements at the time *k*. Vector functions $f_{k}:R^{n_{x}}\to R^{n_{x}}$ and $h_{k}:R^{n_{x}}\to R^{n_{z}}$ are known and $w_{k}\in R^{n_{x}}$, $v_{k}\in R^{n_{z}}$ represent known, mutually independent zero-mean state, and measurement noise, respectively. The solution of the filtering problem is given by the Bayesian recursive relations consisting of two stages: the prediction and the correction:

(4) $$\underset{\mathrm{prediction}}{\underbrace{p(x_{k}|z_{1:k-1})}}=\int \underset{\mathrm{prior pdf}}{\underbrace{p(x_{k-1}|z_{1:k-1})}}\underset{\mathrm{transition}}{\underbrace{p(x_{k}|x_{k-1})}}dx_{k-1}$$

(5) $$\underset{\mathrm{correction}\;(\mathrm{posterior pdf})}{\underbrace{p(x_{k}|z_{1:k})}}=\frac{\overset{\mathrm{predicted pdf}}{\overbrace{p(x_{k}|z_{1:k-1})}}\overset{\mathrm{evaluation}}{\overbrace{p(z_{k}|x_{k})}}}{\underset{\mathrm{normalizing constant}}{\underbrace{\int p\left(x_{k}|z_{1:k-1}\right)p\left(z_{k}|x_{k}\right)dx_{k}}}}$$

At the prediction stage, the pdf is distributed and spread according to the transition model (Equation (2)), which brings more uncertainty to the state estimation regarding the noise $w_{k}$. For the system state estimation $x_{k}$ at the time *k*, the measurements $z_{1:t}$ are required. Depending on the available measurements, one can distinguish stochastic smoothing if t>k, stochastic prediction given t<k, and stochastic filtering problem for t=k. The posterior pdf estimation is computed from the prior pdf, the transition and the evaluation model according to Equations (2) and (3), respectively. This recursive approach to the stochastic filtering enables sequential processing of the measurements, which is suitable for the real-time position estimation. The initial system state is determined at the time k=0 with no available measurements $p\left(x_{0}|z_{0}\right)=p\left(x_{0}\right)$.

### 2.2. Particular Tasks Associated to Pedestrian Dead Reckoning

Various particular methods should be implemented to support the system to serve as a comprehensive indoor positioning system. We review and comment on a few of them, which form the proposed solution, i.e., a step detection, a walk heading calculation, a step length estimation, a vertical localization, and an initial position determination. A supplementary positioning method (e.g., Wi-Fi fingerprinting), in the fusion with the PDR and map constraints, arranges the initial location determination and may update the position estimation, as the PDR error is increasing with the number of estimated positions using inaccurate $L_{t}$ and $\theta_{t}$ values caused by noisy sensor measurements.

#### 2.2.1. Initial Position

The PDR approach estimates a relative position based on a prior estimation. The initial information regarding the absolute position is required for the localization accuracy. Gionata et al. [25] introduced a navigation system for impaired wheelchair users. IMU mounted on a wheelchair provides sensors measurements for PDR and QR codes are used as landmarks with the encoded absolute position. Scanning a QR code may be more user-friendly approach compared to a manual position setup. An outdoor–indoor transition may be detected using machine learning approaches [26]. When the navigation route or the localization initialization starts at the entrance of the building, the initialization with GNSS signal is possible.

Solin et al. [27] initialize the solution based on a first magnetometer reading. Together with other methods, the model converges to a reasonable certainty within a few seconds. If the initial location is unknown, it is possible to setup a stochastic model to represent the prior position as a list of positions with corresponding probabilities. In multiple approaches, where so-called particle filter is utilized, the particles are initialized at random positions with equal weights across the map [21,28].

#### 2.2.2. Step Detection

The detection of a performed step invokes the process of a new position estimation. Typically, the step is detected from acquired accelerometer measurements, and the applied method is influenced by the technique of mounting or holding the device. When the sensors are fixed on the user’s foot, the stance phase of the foot can be easily detected in measurements and the periodic zero velocity updates (ZUPT) are performed to bound the error [29]. Zhang et al. [30] introduced a method describing zero velocity detection with the hidden Markov model, and four states are used to describe the walking motion. Radu and Marina [31] proposed a localization system called HiMLoc for pedestrians holding their smartphones in hand or in a pocket. The authors reference three different types of step detection methods from accelerometer measurements, i.e., peak detection searching for local maximum or minimum in the acceleration magnitude, searching for acceleration values crossing zero value, and the autocorrelation leveraging the repetitiveness of human walking. In that solution, the zero crossing method was applied on smoothed data. Ho et al. [32] applied a fast Fourier transform to smooth the data and proposed a set of step detection rules. Lee et al. [33] proposed a step detection algorithm with the average accuracy more than 98.6% for any combination of considered step modes and device poses. In the project FootPath [34], the steps are detected when the acceleration value falls by at least a given number within a given time window. An additional timeout value prevents multiple steps detection within the same executed step. Brajdic and Harle [35] evaluated the step detection and counting methods for different smartphone placements, and they highlighted the fact that none of the examined algorithm was 100% reliable.

#### 2.2.3. Step Heading

The magnetometer, often in combination with the gyroscope and the accelerometer, provides a framework for the device orientation detection. The measurements may have a drift, and the overall accuracy is influenced by metals and electrical equipment in the building [36]. Kang et al. [37] proposed an improved step heading estimation, where the drawback of the gyroscope is reduced by magnetometer measurements and vice versa. Seo and Laine [38] in their approach determine the device orientation and then count the steps, which enables dynamic changes in the way of holding the device without any significant error in the step detection. Wu et al. [39] introduced a heading estimation method based on a robust adaptive Kalman filtering, which incorporates measurements from the accelerometer, gyroscope, and magnetometer. Moreover, they integrated a model to limit outliers in the measurement data and to resist negative effects of state model disturbances. Ettlinger et al. [40] observed systematic deviations present in the data obtained from sensors. Their research is focused on an analysis of a measure for the reliability (so-called partial redundancies), i.e., how well systematic deviations can be detected in single observations, and the behavior of partial redundancy by modifying the stochastic and functional model of the Kalman filter.

#### 2.2.4. Step Length Estimation

Vezočnik and Juric [41] provided an in-depth analysis of different approaches to the step length estimation. All thirteen considered models are classified to one of four categories: step-frequency-based, acceleration-based, angle-based, and multiparameter. The evaluation was performed for different walking speed values and various device placements: pocket, bag, hand-reading, and hand-swinging. Constants were classified either as universal for all tests or personal tuned for each subject. Experiments using personalized constants gained better accuracy compared to the universal constants. However, the process of constants determination is time-consuming, which may be avoided in some use cases. For personalized constants, the accelerometer-based solutions outperformed the others. For universal constants, the step-frequency-based models obtained the most accurate results. Model proposed by Tian et al. [42] performed best for personalized and model proposed by Weinberg [43] for universal set of constants.

#### 2.2.5. Vertical Localization

The indoor environment introduces a few novel challenges compared to outdoor navigation systems including the positioning and the path planning through multiple floors. Bojja et al. [44] proposed a localization approach for vehicles and pedestrians, where the three-dimensional position is estimated using the particle filter. However, most solutions utilize barometer measurements to detect floor transitions or to obtain the altitude information. Moreover, the position estimation is performed on a single floor. The activity recognition supports the floor change detection by determining the transition method, e.g., stairs, escalators, or elevators. Detecting a less frequent activity, such as using an elevator, may further serve as a landmark to reduce the localization error in PDR by providing more precise ground truth information [20]. Different solutions were implemented for the activity recognition, e.g., deep learning approach [45] or a decision tree proposed by Wang et al. [36], where at the top level the elevator is identified based on a unique accelerometer data pattern. The variance in acceleration measurements separates walking and stairs from escalator and stationary, which are discerned using magnetometer values. Xia et al. [46] highlighted the fact that the height of a floor is not always known. In their solution, multiple barometers were installed in the building to provide reference pressure values. Pipelidis et al. [47] proposed a dynamic vertical mapping system using crowdsourced sensor measurements.

### 2.3. Bayesian Filtering Methods

Indoor localization systems using PDR for the position estimation utilize the Bayesian filtering technique to deal with the uncertainty accumulated by noisy sensor measurements. The error is reduced by incorporating another source of information, typically the map model. Moreover, the filtering may be applied to various particular problems, e.g., the step length model calibration [48] or the activity recognition [49].

Bayesian relations (defined in Equations (4) and (5)) specify only the conceptual solution of the filtering problem. Arulampalam et al. [50] provide an overview of the Bayesian filtering and its implementations, i.e., Kalman filters, particle filters and grid-based methods. As stated by Arulampalam et al., the belief distribution is approximated when certain assumptions for the exact solution calculation are not true, i.e., the analytic solution is intractable. The optimal Kalman filter assumes the posterior density at every time to be Gaussian and the optimal grid-based filter requires the state space to be discrete with a finite number of states, that is not fulfilled in the indoor positioning problem.

Considering the indoor localization, the Kalman filter and the particle filter are the most frequent approaches. In general, the state computation using the Kalman filter or its derivatives has smaller computational demands compared to other approaches. However, if a probability density is non-Gaussian, the Kalman filter cannot describe it well. In ref. [51], the Kalman filter was overperformed by the particle filter in case of an imperfect trajectory obtained using Wi-Fi or with fewer available access points. Table 1 presents an overview of a few published approaches to indoor localization using Bayesian filtering. Use cases and device configurations vary in different solutions. Presently, most of existing solutions utilize smartphones equipped with sensors which provide connectivity and accessibility to the localization system. The particle filter is widely used Bayes filter implementation for the positioning. Nevertheless, different approaches for the system state definition and a fusion with additional techniques are applied to achieve satisfactory results.

### 2.4. Solution Overview

**Figure 1:**
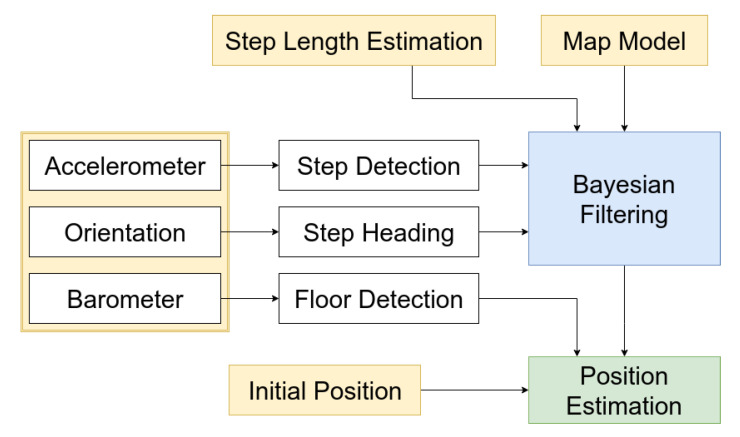
Solution Overview. The aim of the paper is to compare different Bayesian filtering implementations.

The aim of this paper is to evaluate and compare different Bayesian filtering methods, which probabilistically model the uncertainty introduced by noisy measurements. Even though ideal approaches are not chosen for every particular problem, the analysis of the performance with inaccurate parameters is conducive to the overall solution set-up, e.g., how to configure methods to handle underestimated or overestimated step lengths. An overview of our approach is outlined in Figure 1 with the description of chosen methods.

- **Smartphone sensors** used for the step detection and the walk direction determination are the accelerometer and orientation sensors, the magnetometer, and the gyroscope, depending on the device. The barometer is utilized for the floor change detection. We consider only the handheld smartphone or tablet with the user looking at the screen. Sensors measurements are obtained via API provided by the Android operating system.
- **Initial position** is set manually for the evaluation purpose, as it is assumed to be known. However, in deployed application it would be replaced by an approach, which is more user-friendly, such as scanning QR codes strategically placed in the building or possibly using an additional method, e.g., Wi-Fi fingerprinting. When a floor transition is detected, the initial position is set at the entry point to the floor, e.g., elevator doors or a position at the top of stairs.
- **Step detection** is performed on accelerometer measurements, which are smoothed to reduce the noise. A low-pass filter is applied for smoothing and zero-crossing method proposed in [22] is executed to detect steps.
- **Step heading** is obtained using API provided by the operating system. Android operating system calculates the device position from the accelerometer and a geomagnetic field sensor [56]. Bearing values are filtered to reduce the noise.
- **Step length estimation** is predefined for the purpose of this paper and fixed for a single evaluation path. One of the research questions is, how the system is able to handle underestimated and overestimated step lengths. In this case, the step length may be calibrated for a selected user instead of computing the value from measurement data.
- **Floor detection** transition is performed using barometer measurements. The current position is estimated on a single floor. Locations of all stairs, escalators, elevators and other places, where it is possible to change the current floor, are collected in advance. If the transition is detected, the new user position is set to the appropriate location according to the prior position knowledge.
- **Map model** is semi-automatically generated from floor plans by labeling walls manually in the image. Map model provides information whether a position is accessible in the building or there is a permanent obstacle, e.g., walls, places behind walls, and outside the building.
- **Bayesian filtering** probabilistically estimates a dynamic system state, which is restricted to a single floor. We compare different implementations of the Bayesian filtering in this paper.
- **Position estimation** is calculated from the Bayesian filtering state, where the place with the highest belief value is denoted as the estimated position.

### 2.5. Indoor Positioning Using Bayesian Filtering

The indoor positioning can be designed as the Bayesian filtering problem, i.e., the transition and the evaluation functions, the state and the measurement noise, the system state, and the measurement vectors are required to be defined. Some of these settings are analyzed in this paper, as we considered and tested multiple choices. Our research is focused on grid-based implementations of the Bayesian filtering. The main drawback of such filters is the computational complexity, which is growing exponentially with the number of dimensions [23]. Therefore, the system state is preferred to be low-dimensional. The system state vector may consists of more parameters, e.g., velocity and direction, as discussed later. In our case, the state is determined only by the 2D position:

(6) $$x_{k}=\left(x_{k},y_{k}\right)$$

where the $x_{k}$ and $y_{k}$ coordinates may be expressed as longitude and latitude, respectively. However, we prefer to use integers with centimeter-level precision denoting a distance to the reference point in the map. When a step is detected, the posterior system state $x_{k}$ is computed using recursive relations from the state at the time k−1. The current measurement vector at the time *k* may be expressed as follows,

(7) $$z_{k}=\left(\theta_{k},L,A_{map}\right)$$

where $\theta_{k}$ denotes the heading value in degrees and *L* is the detected step length. In our model, we consider the same value of the estimated step length for all detected steps. The parameter $A_{map}$ represents the map model by storing information about the accessibility of positions in the building. Many buildings are formed of regular corridors which are mostly parallel or perpendicular to each other. The map model coordinate system is adjusted that the orientation of the corridors are west–east or north–south. Heading values obtained from sensors are translated with regard to the map rotation, i.e., $\theta_{k}=0$ denotes the direction along the y-axis of the respective floor plan instead of the direction to the north. When a transition between floors is detected, the estimation model is reset (k=0), a new map model is loaded and the current initial state is set according to the entry position.

**Transition** (Equation (2)) is performed using the PDR relative position estimation (Equation (1)):

(8) $$f_{k}\left(x_{k},y_{k}\right)=\left(x_{k}+L\times \sin\left(\theta_{k}\right),y_{k}+L\times \cos\left(\theta_{k}\right)\right)$$

The state noise is a zero-mean white noise with the covariance matrix $cov\left(w_{k}\right)=diag\left(\sigma^{2},\sigma^{2}\right)$. Different standard deviation values σ are tested in our experiments.

**Evaluation** (Equation (3)) is based on the provided map model, as seen in this simplified function:

(9) $$h_{k}\left(x_{k},y_{k}\right)=\left\{\begin{array}{cc} 1 & \;\mathrm{if}\;(x_{k},y_{k})\;\mathrm{is}\;\mathrm{accessible}\\ 0 & \;\mathrm{if}\;(x_{k},y_{k})\;\mathrm{is}\;\mathrm{not}\;\mathrm{accessible} \end{array}\right.$$

Inaccessible positions in the building represent walls, restricted places, permanent obstacles, and, in some cases, places outside the building or the map. However, determining the accessibility only from the location vector is not sufficient in most approaches. The direct PDR transition from an accessible point *A* to an accessible point *B* with a wall between them should be restricted. Related indoor localization systems often incorporate the map model into the transition function. In ref. [5], a navigation graph and a mesh approach are proposed. The transition is performed only along these structures preventing the step transition to intersect any obstacle. In our approach, the transition is accomplished with no restrictions, but at the evaluation stage, the accessibility is determined using both positions—the current one and the previous position, from which the transition was performed. Moreover, the evaluation depends only on the map model. Therefore in our implementation, the measurement noise is omitted, i.e., $v_{k}=0$.

## 3. Grid-Based Filter

The grid-based filter applied on the indoor positioning, extended with some practical improvements, is discussed in ref. [22]. For the purpose of this paper, the brief outline of the method is introduced in accordance with the specified Bayesian filtering formulation and these methods are further elaborated. The enhanced convolution mask computation and the centroid grid filter are proposed as an extension to the referenced work.

### 3.1. Grid Design and Computation

A floor plan is tessellated into a regular grid consisting of $N_{s}$ grid cells $\left\{x_{k}^{i}:i=1,\cdots ,N_{s}\right\}$ or $N_{s}$ isolated points $\left\{{\bar{x}}_{k}^{i}:i=1,\cdots ,N_{s}\right\}$. Typically, ${\bar{x}}_{k}^{i}$ is a center of the grid cell $x_{k}^{i}$. The probability distribution is approximated at these points with associated weights $w_{k}^{i}$, likewise the particle filter:

(10) $$p\left(x_{k}|z_{1:k}\right)\approx \sum_{i=1}^{N_{s}}w_{k}^{i}\delta \left(x_{k}-x_{k}^{i}\right)$$

In this case, the weight or the belief value of a point denotes the probability that the current position is within the grid cell. Probability density after the prediction stage execution is expressed as

(11) $$p\left(x_{k}|z_{1:k-1}\right)\approx \sum_{i=1}^{N_{s}}{\bar{w}}_{k}^{i}\delta \left(x_{k}-x_{k}^{i}\right)$$

where weights ${\bar{w}}_{k}^{i}$ are computed from the prior grid using the transition model:

(12) $${\bar{w}}_{k}^{i}\approx \sum_{j=1}^{N_{s}}w_{k-1}^{j}p\left({\bar{x}}_{k}^{i}|{\bar{x}}_{k-1}^{j}\right)$$

Weights in the posterior state are computed using the predicted weights ${\bar{w}}_{k}^{i}$ and the evaluation model followed by the normalization

(13) $$w_{k}^{i}\approx \frac{{\bar{w}}_{k}^{i}p\left(z_{k}|{\bar{x}}_{k}^{i}\right)}{\sum_{j=1}^{N_{s}}{\bar{w}}_{k}^{j}p\left(z_{k}|{\bar{x}}_{k}^{j}\right)}$$

In Equation (12), the belief value is calculated using a convolution. Convolution masks depend on transition and noise models and define how the probabilities are redistributed during the prediction stage. Moreover, it is possible to precompute the masks for selected values of the direction and the step length to reduce the computational demands during the state estimation. Convolution masks are typically smaller than the entire grid since the transition probability $p({\bar{x}}_{k}^{i}|{\bar{x}}_{k-1}^{j})=0$ for all pairs of points with the distance greater than a single step length plus the maximal noise value. Equation (12) expresses the method that for every point in the posterior grid, the weight is calculated from the set of points in the prior grid according to the mask. The reverse process may be beneficial for the implementation, where the prior grid points are iterated in a loop instead of the posterior grid points. It allows to omit the convolution execution for points with zero or negligible weights. In the implementation, the evaluation (Equation (13)) is performed during the convolution, enabling to improve the usage of the map model. Therefore, the accessibility of a grid cell is not determined only by its position, but also as an accessibility of the transition, i.e., a grid cell *T* is accessible from a grid cell *S* if all cells composing the path from *S* to *T* are accessible. The path is constructed using a line drawing algorithm and precomputed for relative positions of any two grid cells in the mask. Moreover, different grid structures were investigated in the referenced previous work, i.e., the regular square grid and the hexagonal grid consisting of regular hexagons, where the distance between centers of two adjacent grid cells is the same in all six directions.

To sum up a single iteration, when a step is detected, a convolution mask is loaded according to the step length and the direction, the convolution is performed, the grid is normalized, and the new position estimation is found at the grid cell (center point) with the highest belief value. The convolution mask is applied on all grid cells with positive belief values, whereas the accessibility of the path between a source cell and the target cell is verified.

### 3.2. Convolution Mask

**Figure 2:**
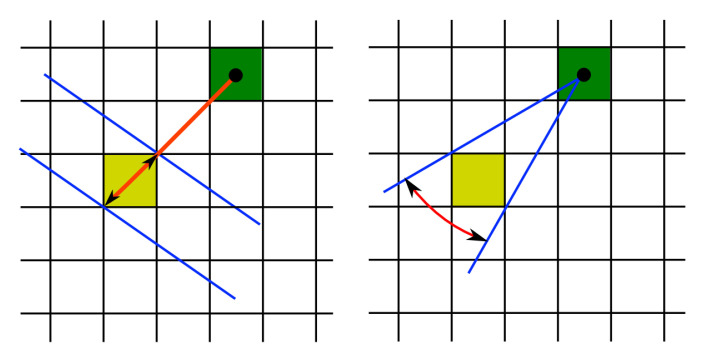
The convolution mask computation. The green grid cell denotes the origin of the mask, and the value for the yellow grid cell is computed using the cumulative distribution function for the normal distribution of the step length or the heading, respectively. The left figure displays the bounds for the step length and the bounds for the heading in the right figure.

Processing a detected step is performed using the convolution followed by the weight normalization. An efficient convolution implementation iterates only over active prior grid cells, i.e., grid cells with positive weight values. It is possible to define the threshold making the cells with their associated weight under the threshold negligible and prevent them from the computation. In this case, the threshold is set to 0. Weights in the posterior grid are computed in the loop using the formula for a pair of indices *i* and *t* denoting the transition between these two grid cells:

(14) $$w_{k}^{t}=w_{k}^{t}+m_{\alpha ,L}^{t-i}w_{k-1}^{i}h\left(i,t\right)$$

where the index *i* denotes a point in the prior grid associated with the positive weight, i.e., $i=\{b\in \left\{1,\cdots ,N_{s}\right\}:w_{k-1}^{b}>T\}$ with the threshold T=0 and the index *t* denotes the target point in the posterior grid. The function h(i,t) returns the value one, if the grid cell *t* is accessible from the grid cell *i*, and it is zero otherwise. The convolution mask $M_{\alpha ,L}$ models the transition and the noise. The mask for a given direction α and a step length *L* is expressed as $M_{\alpha ,L}=\left\{m_{\alpha ,L}^{j}:j=-N_{m},\cdots ,0,\cdots ,N_{m}\right\}$, where the position with the index 0 determines the origin, i.e., the mask position associated with the grid position where the mask is applied. The mask size $2N_{m}+1$ is selected to cover relative positions of grid cells which are affected by the convolution, i.e., the values which are not 0. The value $m_{\alpha ,L}^{j}$ represents the probability of the transition from the origin position (j=0) to the relative position at the index *j*. The value $m_{\alpha ,L}^{0}$ encodes the probability of no movement. The construction of the convolution mask is visualized in Figure 2. The step length and the direction are modeled using normal distribution with the mean at the estimated step length or the measured step heading, respectively, and multiplied by each other to form the value $m_{\alpha ,L}^{j}$. The belief value of a mask grid cell is determined by the cumulative distribution function regarding the bounds of the grid cell. The same approach is applied for the mask computation on hexagonal grids, where only the bounds of a grid cell calculation differs from the square grid. To reduce the computation costs, the masks can be precomputed and stored for specified step lengths and headings. Measured and expected values for the length and the heading are rounded. Therefore, one of stored masks is loaded and applied for the state calculation when a step is detected. In our implementation, relative indices in the mask representing the shifts in the grid are transformed in regard to the full grid size, as the two-dimensional position is encoded in a single index.

A more accurate method for the mask computation is proposed in this work using two layers of grids. A coarse grid is identical with the previous method, where the space is tessellated into grid cells with assigned weights. A fine grid layer has the same structure, but consists of more points, as the distance between two adjacent points is smaller, i.e., the density of points is several times greater. The probability density value is calculated at these fine grid points denoting the probability of the transition from the origin to the given point. The coarse grid weights are expressed as a sum of probability density values of all fine grid points within the single coarse grid cell. The weights are normalized in the grid.

The mask in this approach is defined as a distributing mask, i.e., a value in the mask denotes the probability of the transition from the origin to the given target point. Therefore, the convolution is iterated over the prior grid and the mask determines how the belief value is distributed in the posterior grid. The opposite method defines a mask value as the transition probability from the given point to the origin. The step processing algorithm iterates over the posterior grid cells. This approach is beneficial for the centroid grid filtering method introduced in this paper.

### 3.3. Centroid Grid Filter

**Figure 3:**
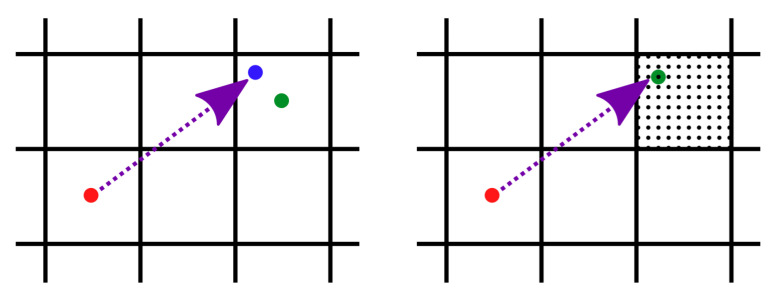
Purple arrow represents the step from the red point. Left figure shows that in the grid filter, the next position is estimated at the green point in the grid cell center point instead of the true location denoted by the blue point. In the centroid grid filter (figure on the right), the next position is chosen from the set of fine grid points.

A rule of thumb for a real-time indoor positioning application is that the position estimation should be completed before the succeeding step is detected. Therefore, it is possible to improve the localization accuracy by increasing the number of all grid points only to some extent. The localization error is influenced not only by noisy inputs, but also by the discrete approximation of the continuous state space in grid-based filters. The proposed approach assumes two grid layers, as in the introduced mask computation. The weight is associated with the coarse grid cell, but the estimated position is not always represented by the center of the grid cell, as visualized in Figure 3.

Formally, the coarse grid $G_{k}$ consists of $N_{s}$ grid cells $G_{k}=\left\{x_{k}^{i}:i=1,\cdots ,N_{s}\right\}$. Every grid cell $x_{k}^{i}$ is associated with the weight value $w_{k}^{i}$ (Equation (10)), and the position estimation expressed as ${\bar{x}}_{k}^{i}$ and a fine grid of $N_{u}$ points $G_{k,i}^{\prime }=\left\{x_{k}^{\prime }i:i=1,\cdots ,N_{u}\right\}$. In this approach, the ${\bar{x}}_{k}^{i}$ is not the center of the grid cell, but selected from the set of points $G_{k,i}^{\prime }$. A so-called centroid of a coarse grid cell $x_{k}^{i}$ is labeled as $c_{k}^{i}$, and it denotes the corresponding index in the $G_{k,i}^{\prime }$, i.e., $c_{k}^{i}=j$ such that ${\bar{x}}_{k}^{i}=x_{k}^{\prime j}$. The fine grid covering the entire state space is defined as $G_{k}^{\prime }={⋃}_{i=1}^{N_{s}}G_{k,i}^{\prime }$. Coarse and fine grid point positions are fixed. Therefore, it is sufficient only to compute weights and select centroids for all coarse grid cells in the posterior state, when a step is detected. Belief values are computed for all coarse grid cells using the formula

(15) $$w_{k}^{t}=\sum_{i=1}^{N_{s}}w_{k-1}^{i}{m^{\prime }}_{\alpha ,L,c_{k-1}^{i}}^{i-t}h\left(i,t\right)\;t=1,\cdots ,N_{s}$$

where h(i,t) is zero, if the transition from the grid cell *i* to the target coarse grid cell *t* is not possible and one otherwise. The value ${m^{\prime }}_{\alpha ,L,c_{k-1}^{i}}^{i-t}$ is obtained from the mask $M_{\alpha ,L,c}^{\prime }=\left\{{m^{\prime }}_{\alpha ,L,c}^{j}:j=-N_{m},\cdots ,0,\cdots ,N_{m}\right\}$. The mask is constructed similarly to the aforementioned method using the fine grid, but the value $m_{\alpha ,L,c}^{i-t}$ denotes the probability of the transition from the centroid position at index *i* to the origin, i.e., the target grid cell where the weight is computed. Masks are created and stored for all combinations of considered step lengths *L*, step headings α and centroid indices *c*. The index of the fine grid position within a coarse grid cell is selected using the weighted centroid approach:

(16) $$c_{k}^{t}=\frac{\sum_{i=1}^{N_{s}}w_{k}^{i\to t}c_{k}^{i\to t}}{w_{k}^{t}}\;t=1,\cdots ,N_{s}$$

where $w_{k}^{i\to t}=w_{k-1}^{i}{m^{\prime }}_{\alpha ,L,c_{k-1}^{i}}^{i-t}h\left(i,t\right)$ is the summand expressed in Equation (15), i.e., $w_{k}^{t}=\sum_{i=1}^{N_{s}}w_{k}^{i\to t}$. The value $c_{k}^{i\to t}$ is precomputed and stored with the mask values determining the target fine grid index, when the probability is transformed from a coarse grid cell *i* to the cell *t*.

To reduce the computational demands on the convolution, the masks are precomputed for selected step lengths and directions. The real values from sensors are rounded and the corresponding mask is applied on the filtering. The weight calculation (Equation (15)) is not performed for all coarse grid cells. Active coarse grid cells with positive weight values in $G_{k-1}$ are iterated, and reachable cells in $G_{k}$, which are not yet computed, are considered as target cells *t*. Another technique to improve the speed of the posterior state computation is resetting the negligible values. Values lower than a selected threshold are set to 0 in the correction phase before all weights are normalized. A less efficient but more stable method keeps a predefined maximum number of active grid cells. Therefore, weights for all cells, sorted by belief values, extending the limit are set to 0. The drawback of such methods is that in some scenarios, especially with incorrect step length estimations, the knowledge of a position may be lost and a recovery method must be implemented based on the history of estimations or other inputs to avoid the uniform distribution of the probability over the entire map as discussed in the evaluation.

To sum up a single iteration, the process after a step is detected is the same as for the aforementioned basic grid filter. However, the convolution phase differs. In our implementation, all grid cells in the prior grid with positive values are iterated to mark grid cells in the posterior grid which are reachable, i.e., the distance is within the mask size. The convolution mask is loaded, the belief values are computed, and the centroid of the particular target cell in the posterior grid is calculated. Optionally, grid cells with positive values in the posterior grid are sorted and reset based on the given maximum number of active grid cells.

## 4. Advanced Point-Mass Filter

The advanced point-mass filter (APM) is a numerical approach to solve Bayesian recursive relations based on the approximation of a continuous state space by one or multiple grids of points. Like grid-based filters, the belief values are computed only on these points. However, the floating grid technique used in APM enables the grid to transform and rotate according to the non-negligible support of the pdf. Therefore, the APM filter is able to focus on the relevant positions with the higher resolution, similarly to the particle filter, but with all advantages provided by the grid approach and the convolution computation. In ref. [57], the full algorithm of the APM is introduced by Šimandl et al. and it is compared with the particle filter applied on a nonlinear state estimation problem. In their evaluation, lower computational demands are achieved without a loss of accuracy. The applicability of the method on the indoor localization problem is investigated in this paper. Specifics of the algorithm and the implementation are commented. Moreover, the evaluation includes the the parameter configuration discussion and the comparison with other filters, especially in terms of the accuracy.

### 4.1. Algorithm Overview

A basic scheme of the adapted method for the two-dimensional indoor localization problem is proposed in this paper. A density function $p(x_{k}|z_{1:k})$ at the time *k* is represented by a grid of $N_{k}$ points $G_{k}=\left\{x_{k}^{i}:x_{k}^{i}\in R^{2},i=1,\cdots ,N_{k}\right\}$, by a set of volume masses for grid cells $D_{k}=\left\{\Delta x_{k}^{i},i=1,\cdots ,N_{k}\right\}$, and by a set of belief values at the points $P_{k}=P_{k|1:k}=\left\{w_{k}^{i}:w_{k}^{i}=p\left(x_{k}^{i}|z_{k}\right),x_{k}^{i}\in G_{k}\right\}$. A set of belief values after the prediction stage of the Bayesian filtering is defined as ${\bar{P}}_{k}=P_{k|1:k-1}=\left\{{\bar{w}}_{k}^{i}:{\bar{w}}_{k}^{i}=p\left(x_{k}^{i}|z_{k-1}\right),x_{k}^{i}\in G_{k}\right\}$. A grid cell volume mass is for the two-dimensional localization interpreted as the area covered by the grid cell. The APM filter assumes the points to be the centers of the corresponding grid cells.

**Initialization:** Define an initial state represented by a grid $G_{0}$, $D_{0}$, and $P_{0}$ for the prior pdf $p(x_{0})$. Then, proceed to following four steps for k=1,2,3⋯.
  (1) **Filtering:** Compute values of the approximate filtering pdf at points of the grid $G_{k}$ using the Equation (5) for the correction stage, for $i=1,\cdots ,N_{k}$:
    (17) $$w_{k}^{i}=c_{k}^{-1}{\bar{w}}_{k}^{i}p_{v_{k}}\left(z_{k}-h_{k}\left(x_{k}^{i}\right)\right)$$
    where $p_{v_{k}}\left(z_{k}-h_{k}\left(x_{k}^{i}\right)\right)$ denotes the evaluation $p(z_{k}|x_{k})$ from the Equation (5) and the normalization constant is defined as $c_{k}^{-1}=\sum_{i=1}^{N_{k}}\Delta x_{k}^{i}{\bar{w}}_{k}^{i}p_{v_{k}}\left(z_{k}-h_{k}\left(x_{k}^{i}\right)\right)$. As the measurements depend only on the map model, its integration is included in the algorithm during the convolution phase likewise the grid-based filter implementation. Therefore, the filtering step consists only of the normalization
    (18) $$w_{k}^{i}=\frac{{\bar{w}}_{k}^{i}}{\sum_{i=1}^{N_{k}}\Delta x_{k}^{i}{\bar{w}}_{k}^{i}}$$
    Optionally, the negligible belief values may be reset to zeros. In this step, the grids are split, if necessary in the multigrid version of the filter and the weights of the grids are computed.
  (2) **Time Update of Grid:** Transform the grid $G_{k}$ to a grid $H_{k+1}=\left\{y_{k+1}^{i}:i=1,\cdots ,N_{k}\right\}$ with the equal number of points using the PDR system dynamics:
    (19) $$y_{k+1}^{i}=f_{k}\left(x_{k}^{i}\right)\;i=1,\cdots ,N_{k}$$
    where $f_{k}$ denotes the state transition function defined in (8). In general, the transition function is allowed to be nonlinear and the grid $H_{k+1}$ may have different structural properties than $G_{k}$. The grid $H_{k+1}$ integrates the state transition noise and covers the support of pdf, i.e., the relevant area, where positive belief values would occur. The local predictive mean and the covariance matrix is calculated and the grid merging for multigrid APM is executed, if necessary.
  (3) **Grid redefinition:** Redefine the grid $H_{k+1}$ to obtain a new grid $G_{k+1}=\left\{x_{k+1}^{i}:i=1,\cdots ,N_{k+1}\right\}$ for the state $x_{t+1}$, incorporating the state noise $w_{t}$, with the same structural properties as the original grid $G_{k}$. The number of points $N_{k+1}$ may be different than the value $N_{k}$ in the preceding state. Compute the number of points, $D_{k+1}$, and rotate the grid according to a computed transformation matrix.
  (4) **Prediction:** Compute values of the approximate predictive pdf at the new grid $G_{k+1}$ using (4) for $i=1,\cdots ,N_{k+1}$:
    (20) $${\bar{w}}_{k+1}^{i}=\sum_{j=1}^{N_{k}}\Delta x_{k}^{j}w_{k}^{j}p_{w_{k}}\left(x_{k+1}^{i}-y_{k+1}^{j}\right)$$
    where $p_{w_{k}}\left(x_{k+1}^{i}-y_{k+1}^{j}\right)$ denotes the transition pdf $p(x_{k+1}|x_{k})$, $x_{k+1}^{i}\in G_{k+1}$ and $y_{k+1}^{i}\in H_{k+1}$. Predictive belief values are calculated using the convolution from corrected belief values $P_{k}$ and grid points determined by the grid $H_{k+1}$.

**Figure 4:**
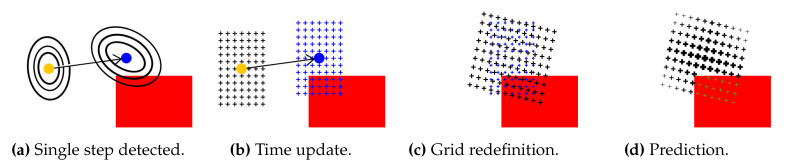
A step is performed from the yellow to the blue point. Circles denotes true pdf (**a**) which is approximated by a grid. Grid *H*_*k*+1_ (blue points) is obtained from the original grid *G*_*k*_ (**b**), which is later (**c**) redefined to the grid *G*_*k*+1_ (black points). The convolution sets belief values (**d**) in respect to the prior grid and the measurements (red area represents a wall; a larger point width denotes a larger belief value).

Figure 4 visualizes a computation of a posterior state. The grid $G_{k}$ is transformed to the grid $H_{k+1}$ and redefined to the posterior grid $G_{k+1}$. The time update stage relocates all points from the prior grid according to the PDR, the measured step heading, and the estimated step length. The posterior grid is further redefined. Moreover, the process includes a calculation of the grid size and the point density in the grid. In the prediction phase, new values for the belief set are computed, based on the prior grid and the map model or measurements in general.

The filtering phase consisting of the normalization and the prediction in the form of the convolution are present also in other grid filters implementations. However, the PDR translation according to the step length estimation and the direction is included in the convolution mask for grid filters. The APM separates these two parts, i.e., all points composing the grid are translated along the vector in the second phase and the convolution models only the noise and is direction-independent. Moreover, the grid points are constructed for the following state (in the third phase) unlike other grid filters where point positions are fixed.

### 4.2. Method Features and Implementation Remarks

The computational time for the APM filtering technique may be reduced by applying particular methods for the grid manipulation. An outline of adopted features of the framework (anticipative and boundary-based approaches, thrifty convolution, and multigrid representation) is provided with additional implementation remarks.

The convolution is a demanding operation of the APM filter and should be implemented smoothly to enable the algorithm to provide position estimations in real-time. The thrifty convolution derives only significant points in the transformed grid $H_{k+1}$ for points in $G_{k+1}$ based on the their distance, i.e., points too far from each other are not included in the convolution computation as the belief gain would be negligible. Moreover, the map accessibility is verified for all grid points. Convolution masks cannot be stored as for other grid filters. However, the calculated values from normal distributions may be cached to speed up the convolution.

The so-called anticipative approach determines the number of grid points and the point mass (corresponding to the grid cell size in grid filters). These calculations are performed during the third phase of the algorithm (grid redefinition). However, for larger scenarios it may be necessary to limit the number of all points. Therefore, this approach is not fully utilized in this work. Moreover, the boundary-based approach detects the area of interest where the grid should be placed. This process is similar to storing active grid cells as proposed in grid-based filters to be considered in the convolution.

To support multimodal distributions, the multigrid representation is introduced by authors of the APM. The basic algorithm is extended with a grid splitting and a merging process. During the first step (filtering) and after the normalization, the grids are examined and a grid is split to two disjoint grids if there are separable areas on the marginal densities. This process should be performed repeatedly until there is no grid to split. However, we found it sufficient to perform this step only once. Two grids may be merged after the PDR transition in the second step (time update of the grid). The criterion for merging two overlapping grids is the Mahalanobis distance between a point and a distribution (mean and covariance matrix are computed for all grids). This method is not covering all situations but it is convenient for the purpose of the method. Moreover, weights are assigned to all grids and these grids are handled independently. The grid weight is computed as a multiplication of the belief sum and the point mass.

## 5. Evaluation

Proposed methods are evaluated on a few sets of experiments. Raw sensor measurements are recorded for each subject with a handheld device while they are walking along the predefined path. Checkpoints with known positions for measuring the localization accuracy are denoted on the floor and indicated in data manually by the user during the experiment. The evaluation of different approaches and configurations is executed afterwards using an offline simulation tool on the same sensor measurements. More than 3000 simulations are considered for this evaluation. The core input files, map models, results, and visualizations are published [58].

Various scenarios were used to support the expectations of proposed approaches and to expose different aspects of the methods. Three different venues are locations of the evaluation. A simple path to verify methods and configuration was set in a faculty building in Slovakia. To evaluate the performance in a real scenario, we used datasets from a competition held at IPIN 2018 (International Conference on Indoor Positioning and Indoor Navigation) in a shopping mall in France (the dataset [59] and the work in ref. [60] available) and IPIN 2019 competition in a research institute in Italy [61].

The main research focus is on the localization accuracy. The overall accuracy may be increased using additional approaches, e.g., Wi-Fi fingerprinting. In the evaluation, we address the following research questions.

- How robust are Bayesian filtering implementations to the inaccurate step length estimation?
- What is the difference in the localization error using introduced versions of grid-based filters and various configuration values?
- What values of standard deviation are suggested to use for considered filters?
- What configuration and version of the particle filter gives the best results and can be chosen as a reference for overall evaluation of considered filters?
- What is the stability of considered filters in terms of using rotated maps or the experiment repetition?
- What is the overall accuracy of the proposed system? Is the performance at the satisfactory level in a new building without any further calibration?

Followed sections discussing the evaluation is organized as follows. A summary of considered methods and scenarios provide an insight to the evaluation background. A methodology and a general performance of the proposed system are outlined followed by the discussion of the parameter configuration for grid based filters, the particle filter, and the advanced point-mass filter. The overall localization accuracy is evaluated on IPIN competition datasets and the discussion of these results concludes the observations for all considered filters.

### 5.1. Considered Methods

The following approaches are applied to estimate user locations especially on predefined checkpoints. These methods are compared with focus on the localization accuracy, computational demands, and their reliability.

1. **Basic Grid Filter** (basic GF) with a two dimensional square-shaped grid introduced in the referenced publication and recapitulated in Section 3.1 and Section 3.2.
2. **Fine Mask Grid Filter** differs from the basic GF in the mask design. The fine mask construction proposed in the Section 3.2 is used for the convolution to estimate the posterior grid state.
3. **Hexagonal Grid Filter** utilizes hexagonal grid cells as proposed in the referenced research and the Section 3.1.
4. **Centroid Grid Filter** with square-shaped grid cells where the position is not attached to the middle point but is chosen from a set of points, as introduced in Section 3.3.
5. **Particle Filter** (PF) is selected for the comparison with grid-based filters. The SIR particle filter implementation introduced by Teammco and Xie [62] is adapted for the evaluation.
6. **Advanced Point-Mass Filter** (APM), proposed by Šimandl et al. [57], is applied on the indoor localization according to the Section 4.

Various configurations of selected methods are investigated. Noise models, i.e., step length and direction standard deviations, and step length estimations are analyzed for all six methods. The accent is on the step length selection. Other parameters are tested with various step lengths to demonstrate how the solutions deal with underestimated and overestimated values.

### 5.2. Scenarios

**Figure 5:**
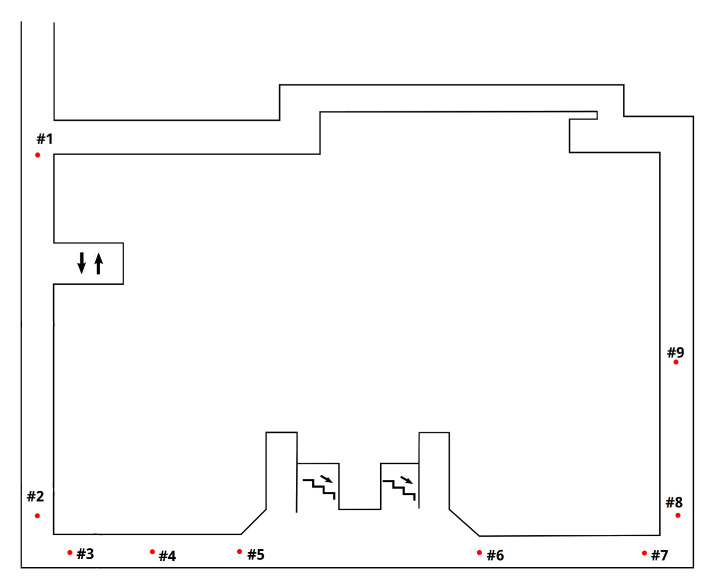
Checkpoint positions in the first scenario. The route starts on #1 and ends on the marker #9 consisting of more than a hundred steps on average. Every subject traveled 85 m along the markers in the ascending order.

The first set of experiments was conducted in the faculty building (Park Angelinum 9, 04001 Košice, Slovakia). Nine checkpoints were placed at positions which support the analysis of the localization error (Figure 5). The scenario consists of a straight walk and two $90^{\circ }$ turns performed in a few steps. Six subjects, mostly students, were asked to walk along the path and mark the checkpoint on the Lenovo tablet held in hand. Before the experiment, their approximate step lengths were calculated using the distance measurement after 20 executed steps (one subject performs the experiment twice), which are expressed in cm {79.1,91.1,79.1,84.7,66.4,78.0,84.7}. The main purpose of this scenario is to evaluate and observe methods and parameter settings in the controlled environment with known subjects and devices supported by external camera recordings. The path is 85 m long and its simple layout, compared to other experiments, makes it suitable for proving concepts, observing tendencies in data, and comparing the influence of some parameters, instead of the complex verification of methods.

The main part of the evaluation is performed on data acquired in the shopping mall Atlantis le Centre near Nantes, France (Boulevard Salvador Allende, 44800 Saint-Herblain, France). Organizers of the IPIN 2018 competition provide the dataset useful for the competition preparation but also for the more objective comparison with other solutions. The dataset consists of testing and validation logfiles. An additional single track for the off-site competition was not used in this evaluation. Every logfile contains between 10 and 24 landmarks where the ground truth position is known and the localization accuracy is computed. These files were used to calibrate parameters of the considered methods and to systematically find the most suitable configuration, especially the major part of testing was executed on the fourth testing path labeled by the logfile T04_01 in the referenced dataset, which is more than 220 m long. The validation logfiles are prepared to score the positioning system. Only a few best configurations based on the testing logfiles are executed in the validation process. The validation measurements were obtained on 6 routes. Every file from 13 available sensor logs has from 10 to 17 ground truth positions. Details regarding the device and the user are unknown.

In the same shopping mall, the on-site competition was held during busy hours on a Saturday afternoon. The more comprehensive path was 800 m long with 70 ground truth locations and includes multiple floors. Sensor recordings were obtained by the author of the paper with Xiaomi MI5 smartphone. Unlike the validation dataset for rating the best configurations, this logfile was used to find the most successful configuration using random search through more possible parameter settings.

The third set of experiments is from CNR Area of Pisa (Via Giuseppe Moruzzi, 56127 Pisa, Italy). Using configurations with the best results from the previous datasets, this part of the evaluation suggests that applied methods are not tailored for the single building scenario but are applicable on different types of maps. The research institute building consists mostly of straight narrow corridors and $90^{\circ }$ turns. The floor transition is possible via multiple stairs and elevators on various positions in the building (elevators were not used in provided data). On the contrary, the shopping mall provides wider corridors with more options in the movement.

### 5.3. General Performance and Observations

The methodology of evaluation on all scenarios is in accord with IPIN competitions [63]. The current user position is computed every time, when the step is detected. If the user steps over the marker sticker on the floor, the button on the device is clicked, the label is inserted into the measurement recording, and stored together with raw sensor data. The position, computed after the checkpoint event is triggered, is compared with the ground truth position for the respective landmark. The Euclidean distance between the horizontal estimation and the corresponding ground truth location is calculated. A floor misdetection is penalized with additional 15 m to the error. The final criterion for the competition is the third quartile (75% percentile) of the position errors on all checkpoints in the single trial.

To demonstrate a general overview of the proposed method, a single trial is chosen from the first set of experiments in the faculty building in Slovakia. The measured step length for the subject was 78 cm. The position error may increase as a result of various factors, e.g., noisy measurements, and incorrect direction estimations and configurations. However, the accuracy improvement may occur due to the map model. Markers #3 and #8 are first checkpoints after junctions. A significant direction change supplemented by map restrictions contribute to the location estimation recovery.

**Figure 6:**
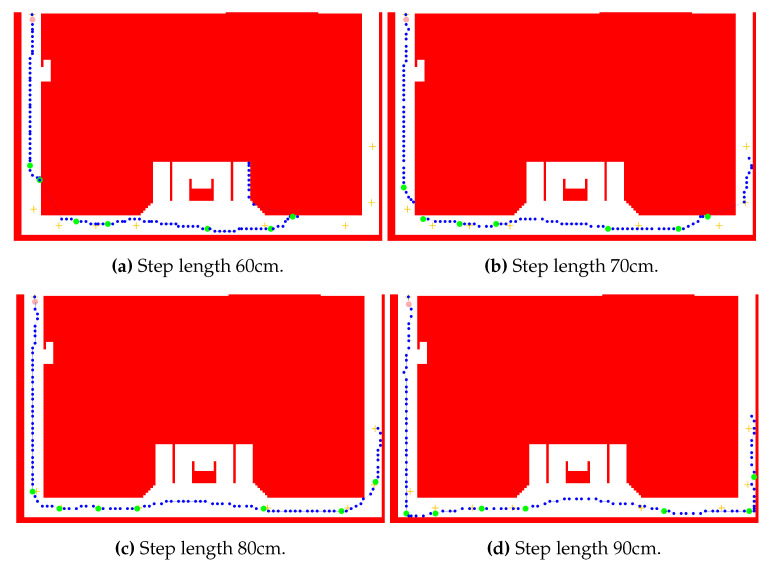
Trajectories of the detected positions using the same sensor measurements, methods, and configurations with different step length estimations. The subfigures display the underestimated step length values (**a**,**b**), the best achieved result (**c**), and the step length overestimation (**d**).

The configuration based on the fine mask grid filter with the best output error was applied using different step length values. Other parameters, which are discussed in next sections, were not changed. Figure 6 demonstrates the performance with selected step length estimations. The step length of 80 cm matches the expected trajectory and produces minimal error. The value of 70 cm underestimates the reality, leading to shorter paths on single corridors compared to the original trajectory. After the direction change, the positions should be predicted outside the accessible area, which is prevented by the map model. If the trajectory of 70 cm steps with given headings fails to continue because of the map restrictions, other convolution mask values become more important and form the position estimations. It is observable mostly after junctions with a larger skip between two consecutive position estimations. In this approach, the step length estimation is not adaptive and therefore the model resumes with the previous value.

The step length estimation of 60 cm enlarges these skips. After the first junction, the position was recovered after a few meters due to the restricted area consisting only of two perpendicular narrow corridors. The second junction caused a significant error as the position is not estimated on the correct corridor. The overestimated 90 cm step length produces similar results to the 70 cm, but skips are replaced by situations with estimated locations accumulated on a very small area along a few consecutive steps. These observations are related to the map model and the performed path. Nevertheless, they support the visual identification of the critical segments when detecting positions along the path. Other parameters affect the ability of the method to deal with these situations. With proper variable values, the system is able to match the correct corridor in this experiment. Third quartile errors in this experiment are 0.5 m for 80 cm step length, 3 m for 90 cm, 4.2 m for 70 cm, and 10.1 m for 90 cm.

### 5.4. Parameters of Grid-Based Approaches

The main research question about the grid-based approach is how the grid design and the position calculation influences the overall accuracy. Location of points, where the belief is computed, depends on the grid cell size and the type of grid (e.g., hexagonal and square grids). Noise models for step length and direction values are included in the selected convolution mask and determine the posterior grid state. The step length and the direction are considered as independent random variables with normal distributions. The mean of the step length distribution is the predetermined step length estimation and the step standard deviation (step SD) is adjustable. The direction has its mean in the measured step heading and the standard deviation (turn SD) is adjustable as well.

**Figure 7:**
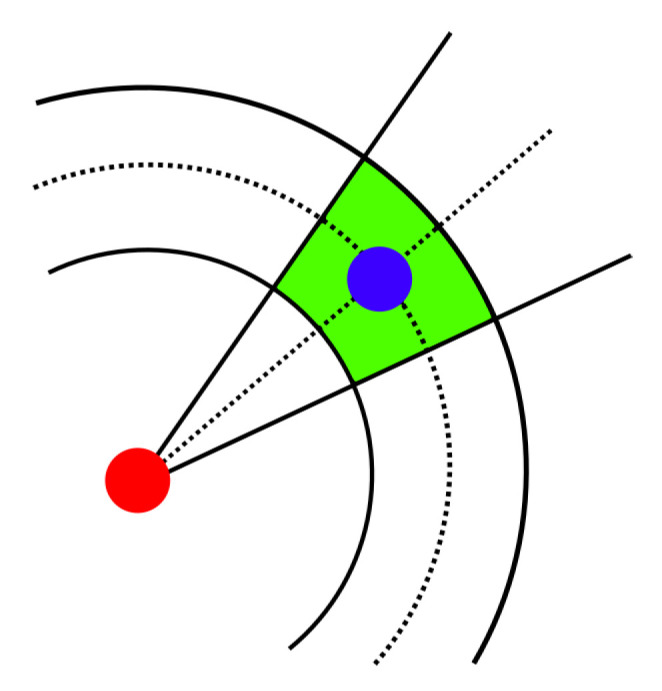
The red point is matched with the current position. The blue dot represents the relative position with the highest probability. Dotted lines marks the mean of the distribution for the step length and the direction. The green area is within the one sigma for both distributions.

The convolution mask introduced in Section 3.2 depends on these two normal distributions. Standard deviations (SD) represent the level of the estimations certainty. Figure 7 displays the area where both the step length and the direction are within one sigma of their distributions. The smaller values of SD give more importance to the mean value and may be less sensitive to invalid estimations. Larger values suppress the significance of estimations, e.g., a large turn SD forms the mask where the step length is more significant and positions may be chosen in all directions with similar probabilities. In the worst case, such configuration may lead to the false trajectory with no possible recovery to the true path. As an example, the fine mask for the step length 80 cm with both SD = 15 has the maximal value 0.3, i.e., the 30% probability of the transition to the respective grid cell. The mask with both SD = 40 has its maximum at 7%. Smaller values of SD increase this number, e.g., both SD = 5, the step length 80 cm gives the maximum value 68% and 90% for the step length 90 cm.

Under certain conditions (e.g., incorrect direction values) the position may be lost. Mostly, in the case of the step length where underestimation with possible posterior positions outside the accessible area occurs, no positive belief value may be left after the calculation is performed and the position is lost. Considering different configurations on the simple path from the faculty building and the T04 logfile from the shopping mall. this phenomenon was detected in 24 runs out of 888. The observation suggests that this problem is present for masks with small standard deviations (under 5 cm). Moreover, the centroid grid filter is more likely to lost its position. The method requires additional computation for the centroids and uses more space to store precomputed masks. In the implementation, more grid cells with negligible values are cut off from the mask compared to other three methods. Other implementations, e.g., the fine mask GF does not fail on the smaller map or with larger standard deviation values. The solution to avoid this phenomenon is to incorporate the no-step in the convolution mask, i.e., set a positive probability to the center position of the mask which is assigned to the prior position estimation. Another option is to implement a backup method to recover from the lost position. If the position is lost, the rollback of the computation is performed and the previous known location is restored. Possibly, the location is restored with a different belief distribution. This option may be beneficial in the presence of an alternative localization method, e.g., the Wi-Fi fingerprinting.

**Figure 8:**
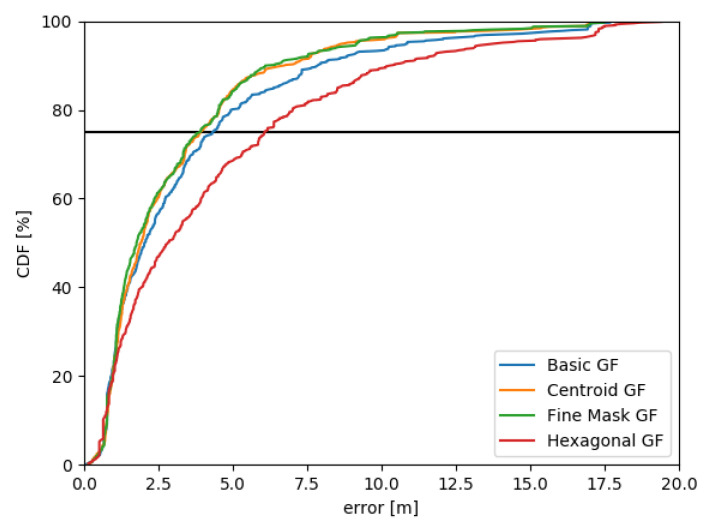
Comparison of lgrid-based filters on all checkpoints in the first experiment scenario with different configurations.

Figure 8 demonstrates the improvement in the grid-based filter design. Errors for all checkpoints from the first experiment were processed, i.e., 9 checkpoints, 7 subjects, 4 methods, 3 step lengths (70, 80, 90), 2 standard deviations (5, 15) excluding all trials where the position is lost, in total 2556 position errors. The most suitable methods for different configurations are the centroid GF and the fine mask GF. The basic grid filter with square cells outperforms the hexagonal grid filter in robustness. However, considering the best of all methods for particular configurations, the fine mask GF got the best result for 30 configurations, the centroid GF for 29 configurations, the hexagonal GF for 19 and the basic GF only for 6 configurations. The hexagonal grid filter achieved satisfactory results for 90 cm step length estimation but accumulated greater error for underestimated step lengths. The overall performance in absolute numbers is discussed together with other methods in this evaluation section. Observations suggests that the centroid GF and the fine mask GF are at the same level, although the centroid GF is more sensitive to the loss of position according to the current implementation.

The influence of step and turn standard deviations was examined on the T04 logfile from IPIN2018 dataset (T04_01). More than 500 outputs were generated with the trajectory visualization, compared, and analyzed. In this case, 160 configurations are considered using the fine mask GF with different step lengths, step and turn standard deviations. Similar tendencies are observable for the centroid grid filter, but the fine mask grid filter was chosen due to full results with no attempts experiencing the position loss. The third quartile of errors for selected configurations vary from 1.9 m to 28.5 m, which supports the significance of the parameters selection.

**Figure 9:**
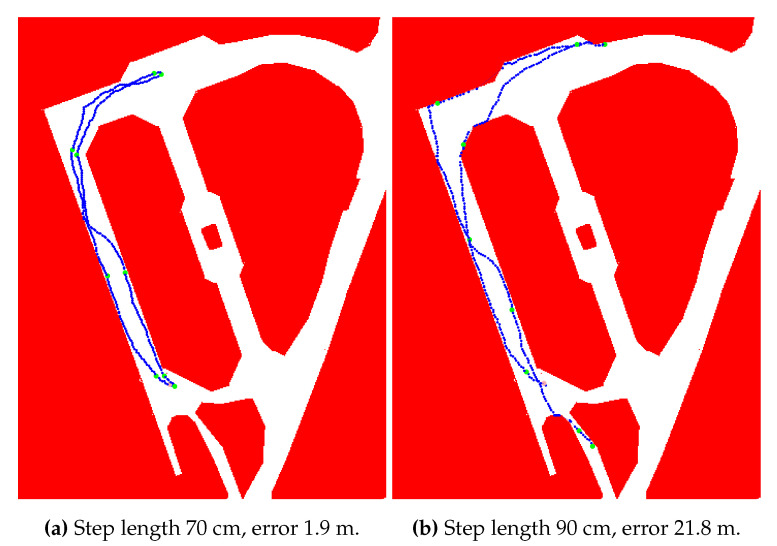
Two trajectories for the T04 logfile. The path in the shopping mall consists of the walking along the corridor, passing to the next corridor to the right, the 180° turnover, and the walking back to the initial position. Both outputs are based on the fine mask grid filter using the step SD 5 cm and the turn SD 25 cm.

Figure 9 visualizes two final paths. This logfile was chosen knowingly to limit the impact of the map model. The ending position is not bounded by a wall, therefore the last checkpoint is sensitive to the proper parameters configuration. Results demonstrate that outputs with the third quartile less than 8 m gain the larger error values mostly near the turnover (typically for 60 and 70 cm step lengths). However, outputs with the overall error greater than 8 m consistently produce the maximal error at the last position (80 cm and 9 cm step lengths). The real step length is unknown, even though the subsequent analysis suggest its value to be 65 cm.

Different values of the step standard deviation does not significantly change the error in contrast to the step length and turn SD. In general, smaller step SD values results to lower error for more accurate step length estimation (60 cm and 70 cm in this case). Considering all configurations, the value 15 cm is a reasonable choice according to the output errors.

**Figure 10:**
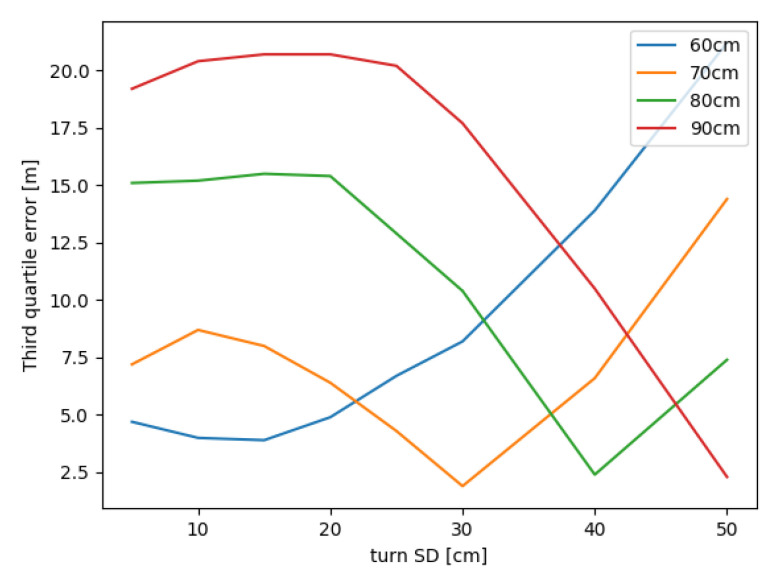
Third quartile of errors for four different step lengths and five turn standard deviations based on T04 logfile with the fine mask GF and step SD = 15.

Figure 10 demonstrates the standard deviation of the direction. As discussed before, small SD values concentrates the belief values on a single or a few positions in the convolution mask. Therefore, the correct step length estimation is essential for the satisfactory output. Large SD values spread probabilities in the mask producing more grid cells with similar positive values even in opposite direction of the current heading. Surprisingly, good results were achieved with turn SD = 50 cm and overestimated step length 90 cm. Nevertheless, these experiments recommend to use turn standard deviation up to 30 cm. Smaller values may be useful in case of the known step length.

**Figure 11:**
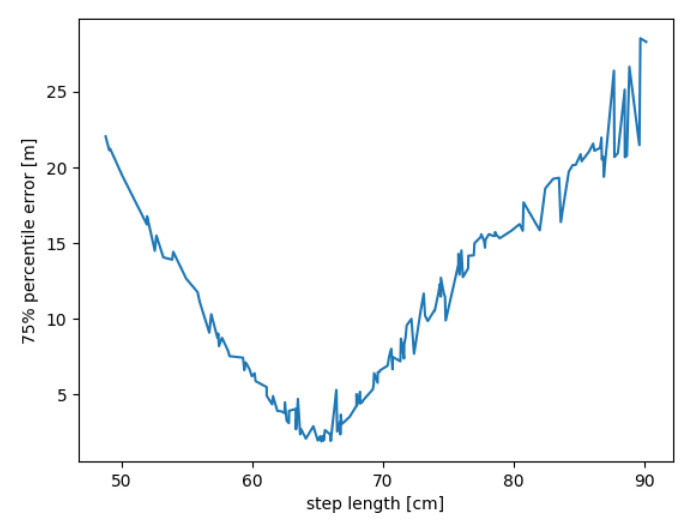
Step lengths are computed as means from all distances between two consecutive steps along the same path. It is not trivial to find the proper configuration for mean step length.

The mean of all distances between two consecutive position estimations was calculated for each configuration output. Figure 11 presents the relation between these mean step lengths and the third quartile of errors. Based on this analysis, it is possible to estimate the true step length which is approximately 65 cm. However, the proper configuration for the given mean step length is not straightforward.

These experiments focus on the convolution mask design and the normal distribution standard deviations analysis. The grid cell size was selected to 33 cm. First attempts operated with 30 cm, then the value changed to 33 to simplify the centroid grid filter calculation using the fine layer of 11×11. The analysis of the grid cell size was not performed and it is still open to further investigation. Another aspect of the evaluation is the map model. The faculty building and the research institute comprise regular corridors which are mostly parallel or perpendicular to each other. The map model coordinate system is adjusted that the orientation of the corridors are west–east or north–south. Direction values obtained from sensors are translated with regard to the map rotation. The analysis of the map rotation influence was performed on the faculty building. The same map rotated by $45^{\circ }$ was created. Based on previous results and observations, the most suitable configuration was selected for each trial. Results for grid-based methods (basic GF, fine mask GF, hexagonal GF, and centroid GF) were compared on the default and the rotated map. The maximum difference on a checkpoint was 19× under 1m and 25× under 2 m out of 28 configurations. Overall, the maximum difference was 6.49m, but under 1m on the marker corresponding to the third-quartile. In total, the final error was worse on the the default map 12 times and 16 times on the rotated map. The situations with better results for the rotated map were observed mostly for the same logfile (subject #6) and for the basic grid filter (6 out of 7). In general, the particle filter and the advanced point-mass filter are map rotation independent. However, the grid tessellation is used for the final position estimation in our implementation. Nevertheless, the differences in the error are not significant.

### 5.5. Particle Filter

Various approaches based on the particle filter contain the current step heading in the Bayesian filter state. Proposed grid-based methods insist on using a minimal number of state parameters, as the complexity and computational demands grow significantly in that case. On the contrary, particle filter may be operating with richer state description. The real time calculation could be guaranteed by reducing the number of particles. This evaluation includes the particle filter with the direction in the system state and with only 2D position for more thorough comparison with introduced grid-based methods. When a step is detected, positions of all particles are translated according to the PDR vector. Moreover, the randomness is introduced by a random movement of the particles typically followed by the weight update and resampling.

**Figure 12:**
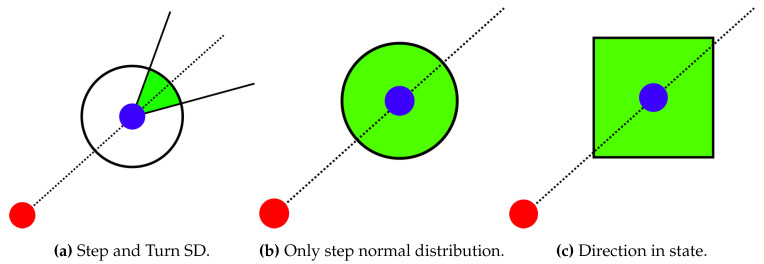
The particle is moved from the red point to the blue point. Afterwards, the particle is transited according to the normal distribution. In panels (**a**,**b**), the direction is not included in the system state.

Figure 12 illustrates the computation in the considered particle filter implementations. In the particle filter with only 2D position as the state, the particle displacement is calculated from the normal distribution of the step length with the mean zero and the step SD standard deviation and the direction normal distribution with the respective parameters. The green area in Figure 12a covers possible positions, where one sigma intervals of both distributions intersect. Another implementation in Figure 12b omits the direction normal distribution and each particle is displaced in a random direction according to the step normal distribution. The green area describes its one sigma interval. Experiments with different configurations suggest no distinct advantage of one of these two versions. Therefore, we use the particle version from Figure 12b as it matches the advanced point-mass implementation.

The particle filter with the position and the direction in the state (inspired by the work in [62]) calculates the random displacement for every parameter in the state separately from the uniform distribution (within the green are in Figure 12c). Moreover, the particle direction value is changed by a random value. The referenced particle filter implementation turns each particle randomly by a given angle to the left or to the right and then performs the movement by the expected step length. Our system does not compute the turning angle but the absolute direction. Therefore, better results are achieved in our evaluation with the proper turning angle with no randomness as calculated directly from the sensors.

The particle filter is a stochastic implementation and the particles are displaced randomly during the posterior state calculation. However, all proposed grid-based filters are deterministic, i.e., repeating the computation with the same configuration produces the same result. Therefore, the part of the evaluation for the particle filter was performed repeatedly. Experiments on the smallest scenario in the faculty building suggests that greater variation in the errors with the same configuration is when the output error is larger. The relative variation for proper configurations achieving sufficient error was under 5%. Considering the shopping mall sensor recording T_04 and 30 different configurations, the relative variation for the same configuration was 12× under 10% and 25× under 25%. Outliers are observable with inaccurate parameter settings.

Different configurations were verified on the same datasets as grid-based approaches (simple path in the faculty building in Slovakia and T04 logfile from the shopping mall). A fixed amount of 1000 particles was set for all experiments which was later increased for the overall accuracy evaluation. Small values of the step standard deviation step SD = 5 lead to position loss and require location recovery. In the faculty building scenario with a relatively small map area, it is expected for the particle filter to outperform the selected grid-based filter for some configurations. The discretization of the space has bigger impact on the final result. The particles are able to model the path with more precision. Tested values between 15 and 25 are not as robust to the incorrect step length as observed in grid-based approaches. Considering the shopping mall scenario and different values for the step length, the best results were obtained with 65 cm step length and step SD = 15 with the average third quartile 3.4 m. Using slightly incorrect step lengths 60 cm and 70 cm, better results were achieved with larger step SD values 30 and 40 (third quartile was between 5.3 m and 6.5 m).

The main goal of this paper is to analyze the grid-based Bayesian approach applied on the indoor positioning. The particle filter is aimed as the reference method for the comparison. Various upgrades may be performed to achieve better results in real scenarios. For instance, Fetzer et al. [5] suggest to calculate the final estimated position at one of the modes of the particle clusters instead of the computed average location from all particles.

### 5.6. Advanced Point-Mass Filter

The process of the posterior state computation in advanced point-mass filter is performed using the same approach as described in the particle filter, where particles are moved according to the step length and the direction and displaced in the random direction by the distance obtained from the normal distribution. In APM, all grids are moved along the PDR vector and subsequently redefined. The convolution is performed to calculate belief values for all grid points. Only the step length normal distribution with the mean zero and the step standard deviation as the parameter is required for the convolution. This method provides more parameters for the configuration. A value *A* defines the non-negligible support of the state noise (the value A>3 is recommended). In our implementation, the value A=4 is used. Greater values enlarges the area covered by the grid during the grid redefinition and considering the same number of the points, the distance between adjacent points is larger, i.e., the resolution is less detailed.

Another parameter, γ, determines the number of points in the grid. The value is recommended to be γ<1. The value γ=0.5 in this implementation produces the spacing in the grid on the level of the given step standard deviation. Smaller values expand the amount of grid points. A grid covering larger areas requires smaller number of points to be able to finish the computation in sufficient time. However, large γ values also reduce these points in grids covering small areas, leading to the larger spacing and thus reduced precision. Therefore, we introduced a threshold for the maximum number of points in a single grid. The amount of grid points is computed automatically using γ=0.5 and for large grids the upper bound on the grid size is set. Therefore, the spacing between points is increasing instead of the number of points in large grids which is necessary for the real-time computation. Moreover, a large grid models the current situation more inaccurate, but it is preferred avoiding the true path loss over the high resolution or the centimeter accuracy.

The value *K* defines the threshold for merging two grids based on their mahalanobis distance. The value K≥3 is recommended. The evaluation on the T04 logfile from the shopping mall demonstrates the influence of this parameter on the grid count and the split or merge grid events. The path consists of 389 detected steps. Using K=3, the maximum number of grids at one iteration was 20 (between 8 and 13 grids for 60% of iterations). The split grid event occurred 565 times (79% of iterations) and merging event 541 times (65% of iterations). Using greater value K=8, two grids are more inclined for the merge. The maximum number of grids was 8 (between 2–4 for 68% of iterations). The split event occurred 124 times (25% of iterations) and merging event 116 times (22% of iterations). The overall accuracy was better for the K=8 (third quartile 3.8 m) than K=3 (third quartile 6.4 m). Other observations supports the tendency to have less grids with larger *K* value which leads to less splitting and merging and the number of points in total for the convolution, even though the difference in the error is not so significant.

In this scenario, the configuration is sufficient for the real time computation. Depending on the implementation, it may be required to reduce the points in grids or to increase the γ for larger scenarios. The largest experiment from the on-site IPIN 2018 competition consists of 882 steps on a few floors. The transition between floors restarts the algorithm with a new initial position. The main segment on a single floor consists of 497 steps. Using the K=3 and the single grid size limit 45×45 points, the maximum number of grids was 30 (only 5 iterations with more than 25 grids). The maximum 60 grids was observed using the grid size limit 23×23 which also leads to less accuracy. Our observations suggests to increase the γ and *K* to achieve the real time computation. Another option is to reset negligible values which is a feature of the algorithm we did not apply in these experiments.

**Figure 13:**
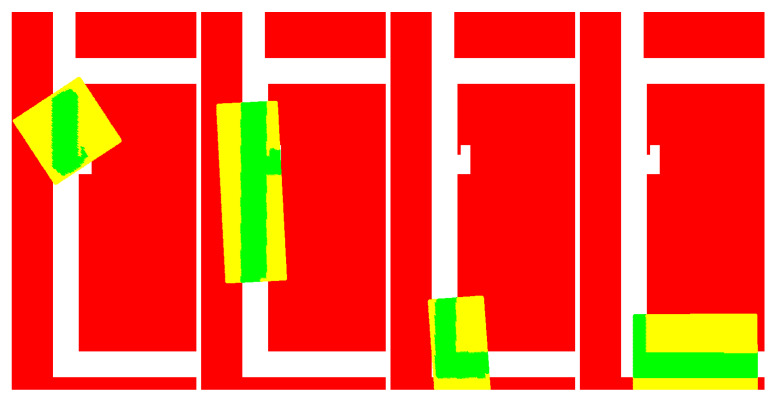
Example of the APM performance. A single grid is placed and rotated with respect to the map and the position estimation certainty. White color denotes accessible areas. Green points in the grid have positive belief values. Yellow points are placed on inaccessible locations.

Smaller values of the step standard deviation (less than 10) often cause position loss similarly to other methods. The recovery solution used in our implementation initiates a new small grid on a position of the last location estimation if belief values in all grids are zero. Similar to the particle filter, better results are obtained with greater values of the step standard deviation (more than 20). Figure 13 demonstrates the performance of the APM algorithm with the ability to focus on selected areas on the map.

### 5.7. Overall Localization Accuracy

The evaluation consists of three venues: the faculty building in Slovakia for the brief verification of selected parameters and methods, the shopping mall in France with datasets from the IPIN 2018 competition, and the research institute in Italy with datasets from the IPIN 2019 competition. For the final comparison of all proposed methods, the evaluation on three scenarios in the last two buildings is executed with the goal to find the best result for every proposed method.

A validation subset of the IPIN 2018 dataset is selected to find the overall accuracy. The main criterion is the third quartile of errors on all checkpoints among all files in the dataset. A few configurations were applied based on the recommendations and observations discussed in previous sections. Moreover, the final estimated path was visualized and critical segments were identified leading to the verification of updated configurations. Table 2 specifies the configurations for the best results, and Table 3 describes the performance of the best configuration on particular validation paths.

Another validation set is present in the IPIN 2019 dataset. The main aim of this evaluation is to prove the map independence. The methods were tuned based on the sensor measurements and the map model of the shopping mall. The research institute differs from the shopping mall with the presence of narrower straight corridors and multiple directions available on junctions. No further result analysis was performed. Only a few best configurations from the first overall evaluation were selected and applied on this dataset. Table 4 describes the best results for all files in the dataset and for all methods and their configurations and Table 5 demonstrates the third quartiles for all particular validation paths.

In all experiments, the configurations were selected systematically. The last experiment was performed on the sensor measurements logfile from the IPIN 2018 on-site competition recorded by the author (with the expected step length 90 cm). The path consists of 70 checkpoints and the duration was more than 10 min. A random search approach was applied to find the best configurations, i.e., the parameter were selected randomly respecting given upper and lower bounds. In total, 773 configurations for all methods were evaluated. This path is more challenging than single paths in validation datasets and provides the comprehensive scenario for the indoor positioning. The best results and configurations for all proposed methods are summarized in Table 6.

The considered particle filter is using 2000 particles and the state is without direction. The advanced point mass filter uses the limit 45×45 points in the single grid.

### 5.8. Discussion of Results

Considering the last evaluation experiment on the IPIN 2018 on-site track, five performances obtained third quartile of the error below 6 m out of 773 runs (2× hexagonal GF, 3× centroid GF). The average of the third quartiles for all used configurations are 27 m for fine mask GF, 26 m centroid GF, 31 m basic GF, and 35 m hexagonal GF which supports the previous observations (Figure 8). The particle filter reached 39 m and the APM 12 m (less configurations with underestimated step lengths were used for these two methods). Considering validation datasets results, tendencies for all methods are observable, e.g., greater errors on the same path V4.1 and V4.2 (Table 3) as it was the longest path with the most freedom in movement with no narrow corridors.

The hexagonal GF achieved the best result in the last experiment, even though the median was the worst among these best runs. Another good result is V02 in IPIN 2019 validation dataset. However, other results demonstrate that the filter is not robust enough to handle invalid step length estimations compared to other filers. The fine mask GF has the same grid design as the basic GF and only exceptionally it does not outperform the basic GF. An example of such event is V2.1 in IPIN 2018 validation dataset. Nevertheless, the visual analysis of the trajectories does not show any particular reason for the exception. We may consider the fine mask GF as the improved version of the basic GF. The centroid GF achieved best results in the last experiment for various configurations. Top 10 performances include 6 with this method. This method is stable and robust even though the position loss occurs with very small standard deviation values (11× out of 160 configurations, other two position loss situations were for the hexagonal GF).

The particle filter obtained the worst results in general. Visualization of trajectories indicates that the position estimations may be improved as the paths are not smooth and zig-zag sequences are observable. Moreover, the filter was not robust enough to handle multiple corridors on junctions resulting to the great error on V05 logfile from IPIN 2019 validation dataset.

Advanced point mass filter did not outperform other methods according to the third quartile of the best performance criterion. However, in the last experiment there were performances with greater third quartile but with the best median of all runs (2.3 m), mean (3.8 m), and 90th percentile (8 m). Moreover, visualization of the paths in this experiment shows trajectories more similar to the real path on the largest straight segment (almost 200 m) as other methods more often tend to have positions close to the walls. One of the main observation of the APM is the ability to track multiple paths. The main path is formed by the positions calculated by the PDR sometimes corrected by the map model. However, when the main path fails, the APM is able to reconstruct the *plan B* position easier and on larger distance compared to other filters due to the multigrid implementations. The particle filter may suppress these positions by resampling and grid-based filters often cut off negligible values to provide real time computation. This feature of the APM is the advantage as seen on V01 in Table 5 but also the drawback as seen on V4.2 in Table 3. The APM method requires more elaboration to find the best configuration, e.g., in terms of number of grids and their spacing, to serve as the best method for the Bayesian filtering on the indoor positioning. In general, these evaluation results demonstrates the applicability of grid-based methods as alternative to the widely used particle filter.

## 6. Conclusions

In this paper, we investigated grid-based implementations of the Bayesian filtering. On the contrary to the commonly used particle filter, these methods are deterministic and utilize the convolution for the posterior state computation. Four versions of the grid-based filter were introduced: the referenced basic grid filter and the hexagonal grid filter, the fine mask grid filter with improved mask computation, and the centroid grid filter using two layers of grids. Moreover, the advanced point-mass filter was proposed connecting strengths of the grid methods (e.g., convolution) and particle filters (e.g., positions not fixed to the grid tessellation).

The evaluation was performed on three sets of experiments using custom measurements and datasets from IPIN competitions. The performance of every method was analyzed and the observations of the parameter configurations were discussed in this paper. The focus is on the overall accuracy measured by the third-quartile of positioning error on checkpoints with known ground truth locations. The parameters were tuned for the shopping mall dataset, where grid-based filters achieved best results between 4.6 and 5.3 m on the validation dataset. The overall results for another venue with no further calibration was between 6.2 and 8.2 m. The best results on the most comprehensive scenario were obtained between 5.5 and 6.4 m.

The overall error may be reduced improving existing approach, e.g., the step length estimation and using other source of information, e.g., Wi-fi fingerprinting. Therefore, as further investigation, we shall focus on the sensor fusion using these Bayesian filtering implementations. Moreover, the influence of the grid cell size, the total number of grids or particles and the time required for the computation is not present in this paper.

## Figures and Tables

**Table 1:** A comparison of a few selected indoor positioning systems in terms of considered device, Bayesian filtering implementation, state representation, and other techniques applied to increase the localization accuracy.

Year & Publication	Device	Bayes Filter	State	Additional Techniques
2019, Lu et al. [52]	chest-mounted IMU	particle filter	position, scale, heading correction	map matching
2019, Xie et al. [53]	robot with camera	unscented Kalman filter	position, velocity, ratio value	visible light
2018, Fetzer et al. [5]	smartphone	particle filter	3D position, heading	Wi-Fi, floor detection, activity recognition, navigation mesh
2015, Chen et al. [20]	smartphone	Kalman filter	2D position	Wi-Fi, landmarks
2015, Xu et al. [54]	smartphone	particle filter	2D position	luminaries
2014, Bojja et al. [44]	smartphone	particle filter	heading angle; East, North and vertical coordinates	3D map matching, moving maps, on-board diagnostics in a vehicle
2013, Radu & Marina [31]	smartphone	particle filter	position, PDR component (activity, distance & compass deviation)	activity classifier, Wi-Fi, map, landmarks
2012, Rai et al. [55]	smartphone	augmented particle filter	2D position, stride length, heading offset	Wi-Fi, map

**Table 2:** The best overall result for every considered method on the IPIN 2018 validation dataset.

Method	Result	Step Length	Step SD	Turn SD
**Fine Mask GF**	**4.60 m**	70 cm	15	30
**Centroid GF**	**4.60 m**	70 cm	15	30
**Hexagonal GF**	**4.92 m**	70 cm	10	10
**Basic GF**	**5.11 m**	65 cm	15	15
**APM**	**5.30 m**	65 cm	30	n/a
**Particle Filter**	**6.18 m**	65 cm	30	n/a

**Table 3:** Third quartiles of errors in meters for considered methods with the best overall result on each validation logfile.

Method	V1.1	V1.2	V2.1	V2.2	V2.3	V3.1	V3.2	V4.1	V4.2	V5.1	V5.2	V6.1	V6.2
**Fine Mask GF**	6.7	6.8	5.8	4.5	3.2	**2.3**	3.5	9.8	8.8	**4.8**	**3.1**	3.1	**3.5**
**Centroid GF**	6.8	6.8	5.8	4.5	3.3	2.4	**3.4**	9.8	8.8	5.1	3.2	**2.9**	4.0
**Hexagonal GF**	**6.2**	**6.2**	4.4	3.6	5.4	**2.3**	3.5	10.8	8.4	3.9	3.9	4.3	5.4
**Basic GF**	**6.2**	**6.2**	**2.9**	4.0	5.4	4.0	5.2	11.5	9.4	3.7	3.5	3.7	4.7
**APM**	**6.2**	**6.2**	4.4	**3.5**	**2.6**	3.0	4.8	8.6	37.7	7.8	3.4	4.6	4.3
**Particle Filter**	8.5	9.5	5.5	4.5	3.9	3.7	6.2	**8.5**	**7.9**	6.0	5.1	3.5	**3.5**

**Table 4:** The best overall result for every considered method on the IPIN 2019 validation dataset.

Method	Result	Step Length	Step SD	Turn SD
**Fine Mask GF**	**6.23 m**	70 cm	15	15
**Hexagonal GF**	**7.09 m**	70 cm	15	10
**Centroid GF**	**7.27 m**	70 cm	15	30
**APM**	**7.49 m**	70 cm	30	n/a
**Particle Filter**	**8.01 m**	70 cm	30	n/a
**Basic GF**	**8.21 m**	70 cm	15	15

**Table 5:** Third quartiles of errors in meters for considered methods with the best overall result on each validation logfile.

Method	V01	V02	V03	V04	V05	V06	V07	V08	V09
**Fine Mask GF**	17.1	12.5	21.1	8.4	**7.6**	5.5	**1.3**	**1.7**	5.6
**Hexagonal GF**	21.7	**8.3**	21.0	8.5	9.0	6.3	3.5	3.7	5.9
**Centroid GF**	25.5	12.3	**5.4**	5.3	8.0	8.2	2.1	4.1	2.9
**APM**	**8.3**	14.1	14.5	8.4	7.7	6.9	2.1	2.6	3.7
**Particle Filter**	88.3	19.6	6.9	**6.2**	57.9	**5.2**	2.2	2.1	4.8
**Basic GF**	25.8	12.5	21.1	8.6	10.3	8.2	2.6	3.2	**2.8**

**Table 6:** The best results for considered methods on the IPIN 2018 on-site competition track. The mean, median, and 90th percentile are listed together with the third quartile of errors on 70 checkpoints.

Method	75th Perc.	Mean	Median	90th Perc.	Step Length	Step SD	Turn SD
**Hexagonal GF**	**5.46 m**	4.38	4.06	8.51	90 cm	5	5
**Centroid GF**	**5.68 m**	4.14	3.52	8.43	90 cm	5	15
**APM**	**6.03 m**	4.48	3.45	9.32	92 cm	44	n/a
**Fine Mask GF**	**6.13 m**	4.16	3.10	8.80	90 cm	25	25
**Basic GF**	**6.37 m**	4.60	3.74	9.21	90 cm	5	10
**Particle Filter**	**7.00 m**	4.79	3.65	9.33	88 cm	35	n/a
